# Prediction of the RNA Tertiary Structure Based on a Random Sampling Strategy and Parallel Mechanism

**DOI:** 10.3389/fgene.2021.813604

**Published:** 2022-01-05

**Authors:** Zhendong Liu, Yurong Yang, Dongyan Li, Xinrong Lv, Xi Chen, Qionghai Dai

**Affiliations:** ^1^ School of Computer Science and Technology, Shandong Jianzhu University, Jinan, China; ^2^ Department of Automation, Tsinghua University, Beijing, China

**Keywords:** tertiary structure prediction, ab initio, Rosetta, Monte Carlo, parallelization

## Abstract

**Background:** Macromolecule structure prediction remains a fundamental challenge of bioinformatics. Over the past several decades, the Rosetta framework has provided solutions to diverse challenges in computational biology. However, it is challenging to model RNA tertiary structures effectively when the *de novo* modeling of RNA involves solving a well-defined small puzzle.

**Methods:** In this study, we introduce a stepwise Monte Carlo parallelization (SMCP) algorithm for RNA tertiary structure prediction. Millions of conformations were randomly searched using the Monte Carlo algorithm and stepwise ansatz hypothesis, and SMCP uses a parallel mechanism for efficient sampling. Moreover, to achieve better prediction accuracy and completeness, we judged and processed the modeling results.

**Results:** A benchmark of nine single-stranded RNA loops drawn from riboswitches establishes the general ability of the algorithm to model RNA with high accuracy and integrity, including six motifs that cannot be solved by knowledge mining–based modeling algorithms. Experimental results show that the modeling accuracy of the SMCP algorithm is up to 0.14 Å, and the modeling integrity on this benchmark is extremely high.

**Conclusion:** SMCP is an *ab initio* modeling algorithm that substantially outperforms previous algorithms in the Rosetta framework, especially in improving the accuracy and completeness of the model. It is expected that the work will provide new research ideas for macromolecular structure prediction in the future. In addition, this work will provide theoretical basis for the development of the biomedical field.

## Introduction

Biological macromolecules such as protein, ribose, and polysaccharides are indispensable substances in living systems, and the structural prediction of biological macromolecules is a grand challenge of bioinformatics. The *ab initio* structure prediction of atomic accuracy was realized in the sixth critical assessment of techniques for protein structure prediction (CASP) blind trial by adopting the conformational sampling method combining low-resolution and high-resolution minimum energy function and the Monte Carlo calculation method (Bradley et al., 2010). RNA secondary structure prediction has improved the performance of EA by introducing a quantum computing strategy and achieved high-precision and high-sensitivity prediction (Shi et al., 2020). As a blind experiment to evaluate the RNA tertiary structure modeling, the purpose of RNA puzzle is to find out the capacity and bottleneck in RNA prediction. A grim result in recent RNA assessments is that the accurate prediction rate of noncanonical base pairs is less than 20% (Miao et al., 2017). However, the RNA structure plays an essential role in molecular biology. On the one hand, the function of RNA mainly depends on its structure (Calonaci et al., 2020). Only by understanding the structure can we further explore the function and the relationship between the function and structure. On the other hand, understanding the RNA structures can provide a basis for medical progress, for example, providing the theoretical basis for designing targeted ribosome drugs (Shi et al., 2014), measuring the epigenomic features of each NC RNA type to provide a theoretical basis for human disease research, especially cancer (Boukelia et al., 2020), and providing a new perspective for disease diagnosis and prognosis (Lu et al., 2021).

The RNA tertiary structure refers to the spatial coordinates of all atoms in RNA (3D structure) and the spatial relationship between atoms embodied by the atomic coordinates (tertiary interaction) (Yang and Liu, 2021). Therefore, the RNA tertiary structure prediction needs to predict van der Waals interactions, hydrogen bond interactions, and other tertiary interactions among atoms and needs to predict the spatial coordinates of all atoms. Because the base is planar, the hydrogen donor/acceptor at the base edge can be roughly divided into three pairing edges, namely, Watson-Crick (W), Hoogsteen (H), and Sugar (S). The paired edges also have forward and reverse directionality. Therefore, four bases can theoretically form 12 hydrogen-bonding patterns (Leontis et al., 2002), proving that RNA tertiary structure prediction is complicated. In addition to the fact that the modeling accuracy of the RNA tertiary structure is inversely proportional to the number of nucleotides (Lorenz and Stadler, 2020), The RNA loop contains unusual torsion combinations, extra helpful bulks, and non-canonical interactions, increasing the difficulty of modeling the RNA tertiary structure. However, if the RNA loop is not modeled, it will be impossible to explain evolutionary data or predict molecular chaperones. Therefore, even if the prediction of the RNA tertiary structure is complicated, it is still necessary.

Researchers have conducted a range of research studies on RNA structure modeling, and numerous RNA tertiary structure prediction algorithms have been proposed. Traditional structure prediction algorithms mainly include MANIP (Massire and Westhof, 1998), ModeRNA (Magdalena et al., 2011), RNABuilder (Flores and Altman, 2010), 3dRNA (Zhao et al., 2012), MC-fold/MC-Sym (Parisien and Major, 2008), FARNA (Das and Baker, 2007), FARFAR (Das et al., 2010), and NAST (Boudard et al., 2015). Improved optimization algorithms based on these algorithms have also been produced, such as the direct coupling analysis (DCA) algorithm of nucleotide coevolution based on 3dRNA (Wang et al., 2017). Moreover, the new RNA tertiary structure modeling methods have also achieved good results; for example, Vladimir et al. put forward a graphical model based on the Leontis–Westhof extended base-pair classification (Waldispühl and Reinharz, 2015). In addition, the emergence and development of Rosetta have made it possible for the modeling of biomolecular macromolecules, which is an extensive software suite for macromolecule modeling. Rosetta has been tailored to address various protein design tasks (Schmitz et al., 2021), and through the Rosetta software suite, constant performance improvements have given rise to a greater breadth of structure prediction, such as the docking and design of antibody and antigen models (Schoeder et al., 2021). Therefore, Rosetta can be used as a framework to model the RNA tertiary structure, whereas the RNA tertiary structure prediction methods in Rosetta need to be improved and optimized.

The predicting algorithms of the *ab initio* RNA tertiary structure have two critical points in the Rosetta framework. First, candidate structures are generated by various sampling methods. Then, the high-resolution energy functions are used to evaluate the generated candidate structures, e.g., Rosetta Energy Function 2015 (REF15) (Alford et al., 2017), a recently updated free energy function. The structure with the lowest free energy or a high score is used as the predicted structure, which is compared with the native conformation to evaluate the performance of the method. Inefficient sampling remains the bottleneck of RNA high-resolution modeling. However, it is impossible to achieve an accurate modeling and test strictly high-resolution energy functions without efficient sampling in the conformational space.

To address the challenge of conformational sampling, Paul Zakrevsky and Rhiju Das put forward a hypothesis called the “stepwise ansatz,” for recursively constructing models by adding residues one at a time, enumerating several million conformations for each motif, and covering all build-up paths (Sripakdeevong et al., 2011). Kladwang et al. further pointed out that replacing deterministic enumeration sampling with random sampling will reduce the calculation cost and improve the modeling accuracy (Watkins et al., 2018). To further reduce the calculation cost and improve the modeling accuracy, we assume that the parallel mechanism is realized by using the shared pool when sampling, stepwise ansatz would further enhance the modeling accuracy, and the judgment and processing of modeling results can further improve the modeling integrity. To test this hypothesis, we developed stepwise Monte Carlo parallelization (SMCP) based on the Rosetta software suite framework, which is a Monte Carlo optimization algorithm whose primary moves remain the stepwise addition or deletion moves. Finally, we report that SMCP can significantly improve the computational accuracy and modeling integrity of *ab initio* structure prediction.

## Materials and Methods

### Datasets

Protein Data Bank (PDB) is a special database for the three-dimensional structure of biological macromolecules such as protein and nucleic acid (Qin and Su, 2019). RNA motifs used in the experiment were filtered from the PDB database. The coordinate data of this database not only need to yield good geometry but also be suitable for experimental data (Berman, 2021). RNA motif information is shown in Table 1. In Supplementary File S1, the benchmark datasets are provided in the fasta format.

**TABLE 1:** RNA motif from the PDB database.

Motif	PDB id	Length
5P_j12_leadzyme	1NUJ	15
5P_p1_m_box_riboswitch	2QBZ	15
j24_tpp_riboswitch	3D2V	5
23S_rrna_44_49	1S72	6
L1_sam_ll_riboswitch	2QWY	7
23S_rrna_1976_1985	1S72	10
23S_rrna_2003_2012	1S72	10
J31_glycine_riboswitch	3OWI	7
hepatitis_C_virus_ires_lla	2PN4	15

### Algorithm Design

RNA modeling under the Rosetta framework adopts the mechanism of combining the sampling method with energy function. On the one hand, threads use sampling methods to search conformations, and all sampling methods in the Rosetta framework are based on the Monte Carlo method. On the other hand, the energy function judges the conformations. In addition, different potential energy evaluation criteria are needed in the modeling process. We use the parameter root mean square deviation (RMSD) when evaluating the conformation of the results generated by modeling.

#### Method of Sampling

Inefficient sampling is still the bottleneck of RNA high-resolution modeling. Efficient conformational space sampling is achieved for accurate modeling. The sampling method based on the stepwise ansatz hypothesis solves the problem of conformational sampling accuracy to some extent. Owing to the considerable sampling space, the computational cost of enumerative conformation search is prohibitive, and the random sampling scheme solves the cost problem of conformation search. However, there are other problems in the sampling process.

**FIGURE 1:**
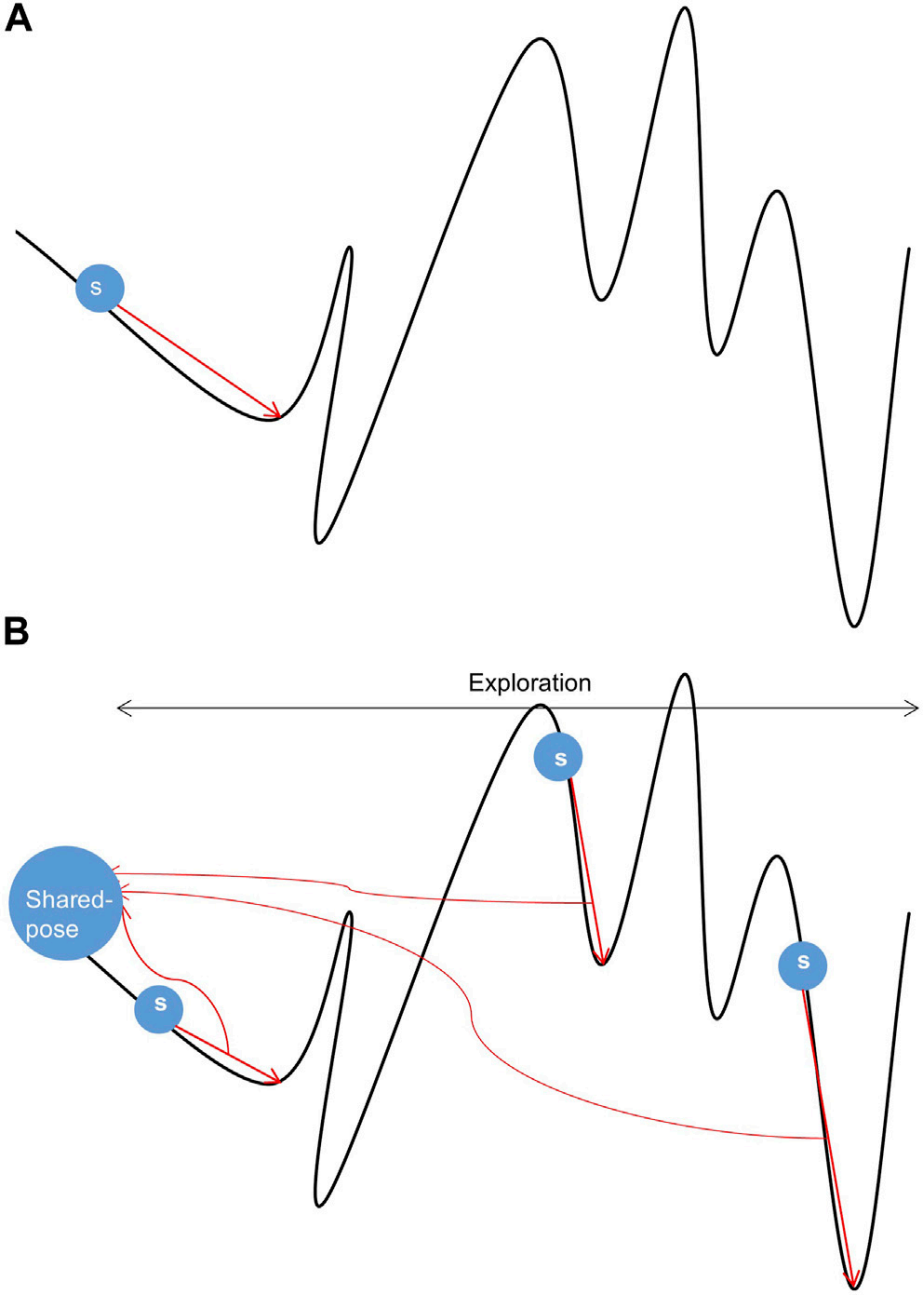
Samples of conformational space. **(A)** Sampling under single-thread. **(B)** Sampling under multi-thread.

The curve trend in Figure 1 (Kolodny, 2005) reflects the energy change in the sampling space. The position of the lowest energy value in the energy landscape map cannot be obtained in advance. Therefore, we can only use energy function and potential energy evaluation to approach the lowest energy infinitely. The energy in the sampling space shown in Figure 1 has a great deal of peaks and valleys, and pseudo-minimum potential energy may be obtained in the sampling process, resulting in low accuracy. Therefore, the existing single-thread sampling method in Rosetta is a significant problem that limits the sampling accuracy.

In Figures 1A, a random search is performed from s while performing conformational sampling. The location of the local lowest energy can be found by the Monte Carlo mechanism. Still, the search ability of a single thread is limited, and it is difficult to cross the energy barrier to find the authentic lowest energy. In Figure 1B, three threads search the same conformation space at different initial positions s. All threads will get a local lowest-energy valley. Comprehensive processing of local conformational samples sampled by all threads dramatically increases the probability of obtaining the actual lowest energy valley in the conformational space, resulting in high-quality samples. In addition, the parallel mechanism has made progress in the field of protein, e.g., using parallel and incremental sample conformational sampling to solve the conformational sampling problem when trying to dock large ligands to proteins (Devaurs et al., 2019).

#### Energy Function

The keys to *ab initio* RNA tertiary structure prediction are to generate candidate structures by the sampling method and to use high-resolution energy function to evaluate the generated candidate structures. Rosetta’s energy function considers more than 30 kinds of energy terms, including fa_stack, ch_bond, fa_elec_rna_phos, and rna_torsion for RNA. All the energy terms with different weights are linearly summed to obtain the final conformational energy value. On the Rosetta framework, the components of all energy functions are the same, and the difference lies in the distribution of weights, i.e., different energy functions have different emphases on influencing factors.

#### Potential Energy Evaluation

The energy function scores the candidate structures to obtain the potential energy value of the conformation. In the process of conception selection, the evaluation of potential energy is critical. Under different circumstances, the potential energy evaluation criteria are different. For one thing, when modeling a single nucleotide based on the stepwise ansatz hypothesis, add, add_submotif, delete, and resample operation will be performed randomly on a single nucleotide, and Monte Carlo will choose whether to accept or reject these moves. At this time, the selected potential energy evaluation criteria are potential energy value decrease or potential energy value increment, which is lower than an energy value set by the Metropolis criterion (Ferkinghoff-Borg, 2012). And, for another, plenty of conformations are generated after modeling. Based on the principle that the low energy structure is more stable, the conformation with the lowest potential energy value is selected as the modeling result.

#### RMSD

The structural similarity in the field of structural prediction in bioinformatics is usually measured by RMSD, which measures the difference between the modeled conformation and the native conformation. Apart from being used to predict the protein structure, RMSD can also predict the structure of non-protein molecules. RMSD is an indicator in the structure prediction algorithm (Mohammad and Hakimeh, 2016).

The focus of RMSD calculation is alignment and optimal superposition of structures. Comparing the structures of two conformations means that it is necessary to establish a 1–1 correspondence between equivalent atoms in each conformation. Then, by rotating and translating a structure to find the best superposition, the weight of the sum of squares of the distance between equivalent atoms in two structures is minimized (Coutsias and Wester, 2019). There are many RMSD calculation algorithms, among which the Kabsch algorithm is well-classic (Kabsch, 1976). The functions that calculate RMSD are as follows (Radoslava and Fatima, 2020).

$$RMSD=\sqrt{\frac{1}{n}\sum_{i=1}^{n}\delta_{i}^{2}},$$

where $\delta_{i}$ is the distance between atom i and either a reference conformation or a mean position of n equivalent atoms. Atomic coordinates are usually expressed by Å [Length units, where 1 Å = 10–10 m(×)]. RMSD is equal to 0 for identical structures, and its value increases when the two structures are different. Therefore, a smaller RMSD value indicates that the similarity between the predicting and native structures is low, which means that the modeling accuracy is low. The RMSD calculator is mainly used to calculate rms distances between the molecules. Therefore, rms (Root Mean Square) has the same meaning as RMSD throughout this study.

**FIGURE 2:**
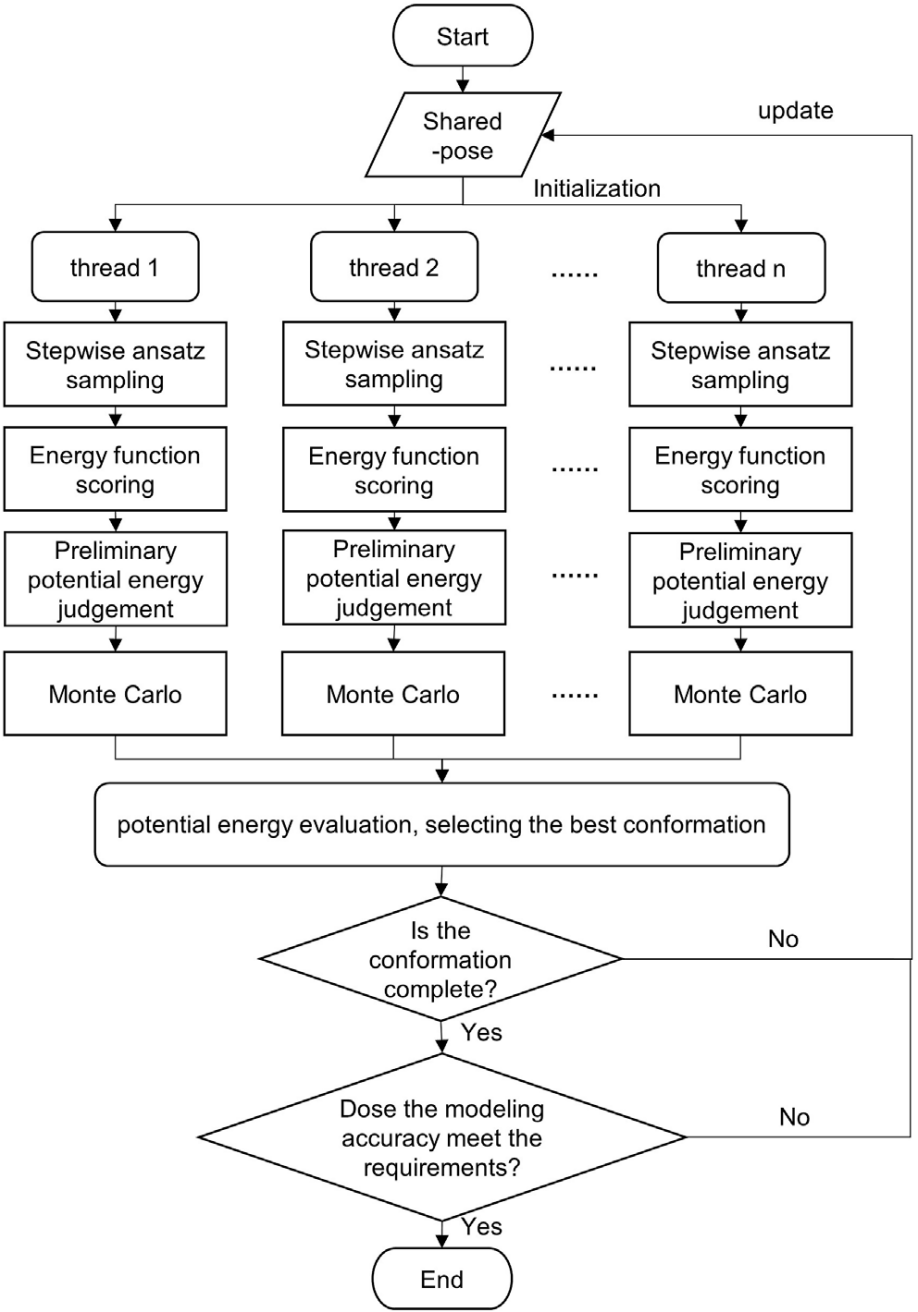
Flowchart of the SMCP algorithm.

In this study, Monte Carlo, stepwise ansatz, and parallel mechanisms are adopted to expand the sampling range by multi-threading. In addition, all candidate conformations are screened by multi-potential energy evaluation criteria, which improves the modeling accuracy. The flowchart of the SMCP algorithm is depicted in Figure 2.

### Proposed Algorithm

The input of the algorithm includes start_pdb file, native_pdb file, fasta file, flags command operation file, energy function *EF*, and the number of parallel threads *n*.

The detailed description of the SMCP algorithm is as follows: first, initialize pose, create n threads, and assign energy functions. Then, each thread is modeled at the same time, and the modeling process mainly includes the following:

1) Conformational sampling. The n threads sample the conformational space at the same time. Based on the stepwise ansatz hypothesis, the operations of add, add_submotif, delete, and resample are performed on a single nucleotide randomly and gradually.
2) Scoring by energy function. The energy value of each conformation is obtained by scoring the energy function.
3) Preliminary potential energy evaluation. According to specific rules, the potential energy is evaluated to select the optimal conformation. The preliminary evaluation criteria are potential energy of the model decrease, or potential energy increment is lower than an energy value standard.
4) Potential energy evaluation. The evaluation criterion is to take the structure with the lowest potential energy in all threads as the local optimal conformation.
5) Integrity and accuracy judgment. The integrity and modeling accuracy of the current optimal conformation are judged. When the modeling conformation is complete and when the RMSD ≤ 2 Å of the predicted structure and the native structure, the predicted structure is considered as the native structure (Townshend et al., 2021); the conformation will be regarded as the final modeling result. Otherwise, the conformation is returned to the shared pose, and then, each thread is reinitialized by using the current conformation for a new round of modeling.

The steps of our SMCP algorithm based on machine learning is as follows:

Algorithm SMCP.

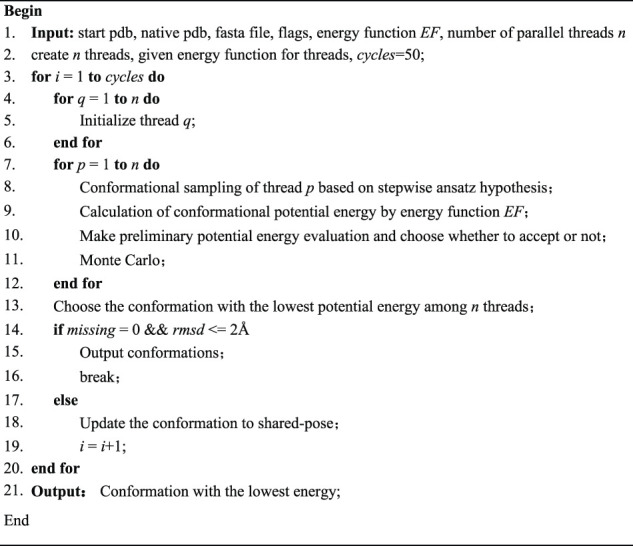

## Results and Discussion

### Parameter Setting

The parameter n indicates the number of parallel threads, and the parameter m indicates the number of Monte Carlo samples. In reality, many threads and Monte Carlo cycles will cause high time costs. Considering the time cost of the experiment and the requirement of modeling accuracy, we set up three parallel threads in the experiment and 10,000 Monte Carlo samples, i.e., n = 3 and m = 10,000. The database materials that we used in this study are based on the research of Wipapat Kladwang et al. Specific materials can be obtained in the experimental section below. In addition, we use the same energy function rna_loop_hires_04092010 as the SWM algorithm, which is to control variables for comparison. The related parameters and weights are described in Table 2.

**TABLE 2:** Parameters of energy function (*rna_loop_hires_04092010*).

Term	Description	Units	Weight
fa_atr	Attractive energy between two atoms on different residues	Kcal/mol	0.23
fa_rep	Repulsive energy between two atoms on different residues	Kcal/mol	0.12
fa_intra_rep	Repulsive energy between two atoms on the same residue	Kcal/mol	0.0029
rna_torsion	Knowledge-based torsional potential	kT^a^	2.9
rna_sugar_close	Penalty for opening an RNA sugar	kT	0.7
hbond_sr_bb_sc	Energy of short-range hydrogen bonds	Kcal/mol	0.62
hbond_lr_bb_sc	Energy of long-range hydrogen bonds	Kcal/mol	2.4
hbond_sc	Energy of side chain to side chain hydrogen bonds	Kcal/mol	2.4
fa_elec_rna_phos_phos	Electrostatic energy (fa_elec) between RNA phosphate atoms	kT	1.05
fa_stack	π-π stacking energy for RNA bases	kT	0.125

a 1kT corresponds to one Rosetta Energy Units (REU).

### Efficient Modeling of SMCP

We randomly performed some moves and chose random positions to manipulate nucleotides instead of listing all possible positions. In addition to add, we also served delete to simulate the instantaneous non-deconstruction of the loop edge nucleotides. Most importantly, we also allowed random selection of the internal freedom of resampling. Based on the conformational sampling method, we carried out multi-thread parallel computation. After the parallel computation, we made a potential energy evaluation, selected the conformation with the lowest free energy in parallel computation, and further evaluated the conformation. To begin with, we judged whether the modeling was complete. Second, we judged the modeling accuracy. After the above evaluation, the final modeling conformation could be obtained if it met the requirements.

Before the more extensive SMCP modeling tests, we first tested the method on 5P_j12_leadzyme, a multi-strand RNA composed of 15 nucleotides. In addition, since SWM is an algorithm with high modeling accuracy under the Rosetta framework, we also used the SWM algorithm to model 5P_j12_leadzyme. We compared the modeling of SWM and SMCP, and the modeling results are shown in Table 3. Several important scoring terms are bound to be highlighted. The first scoring term is *score*, which is the linear addition of the weight given by the energy function and the energy item, which indicates the total atomic free energy value of the RNA structure. A low *score* means low free energy, indicating a stable structure. The second scoring item is *rms*, which indicates the error between the predicted structure and the native conformation. We prefer a small *rms* value on account of it means a high modeling accuracy. The third scoring item is *missing*, and the conformational sampling method is based on the Monte Carlo algorithm, which is randomization and may lead to incomplete modeling. For example, modeling is incomplete if a nucleotide is not successfully modeled. We would like to get *missing* equal to 0, that is, modeling is complete.

**TABLE 3:** Comparing the results of SWM and SMCP.

	SWM	SMCP		SWM	SMCP
score	−35.720	−52.787	fa_stack	−16.279	−25.613
fa_atr	−19.790	−27.994	hbond_sc	−30.093	−37.667
rna_sugar_close	0.974	1.103	hbond_lr_bb_sc	−0.672	0.000
fa_intra_rep	0.311	0.324	hbond_sr_bb_sc	0.000	−0.168
lk_nonpolar	−1.815	−3.402	geom_sol_fast	23.168	23.168
fa_elec_rna_phos_phos	0.566	−0.369	linear_chainbreak	0.978	0.040
ch_bond	−9.296	−14.128	rna_bulge	−4.500	0.000
fa_rep	2.387	3.438	atom_pair_constraint	0.000	0.000
rna_torsion	18.342	20.447	angle_constraint	0.000	0.000
rms	2.136	1.550	missing	1	0

a Both of them are modeling 5P_j12_leadzyme.

Terms in Table 3 show that the *score* value of SMCP is low, which means low energy and more stable structure, *rms* value is low, and *missing* is equal to 0. To sum up, compared with the SWM algorithm, the SMCP algorithm has a higher modeling accuracy and strong integrity.

### *Ab Initio* Modeling on a Complex RNA Benchmark

**FIGURE 3:**
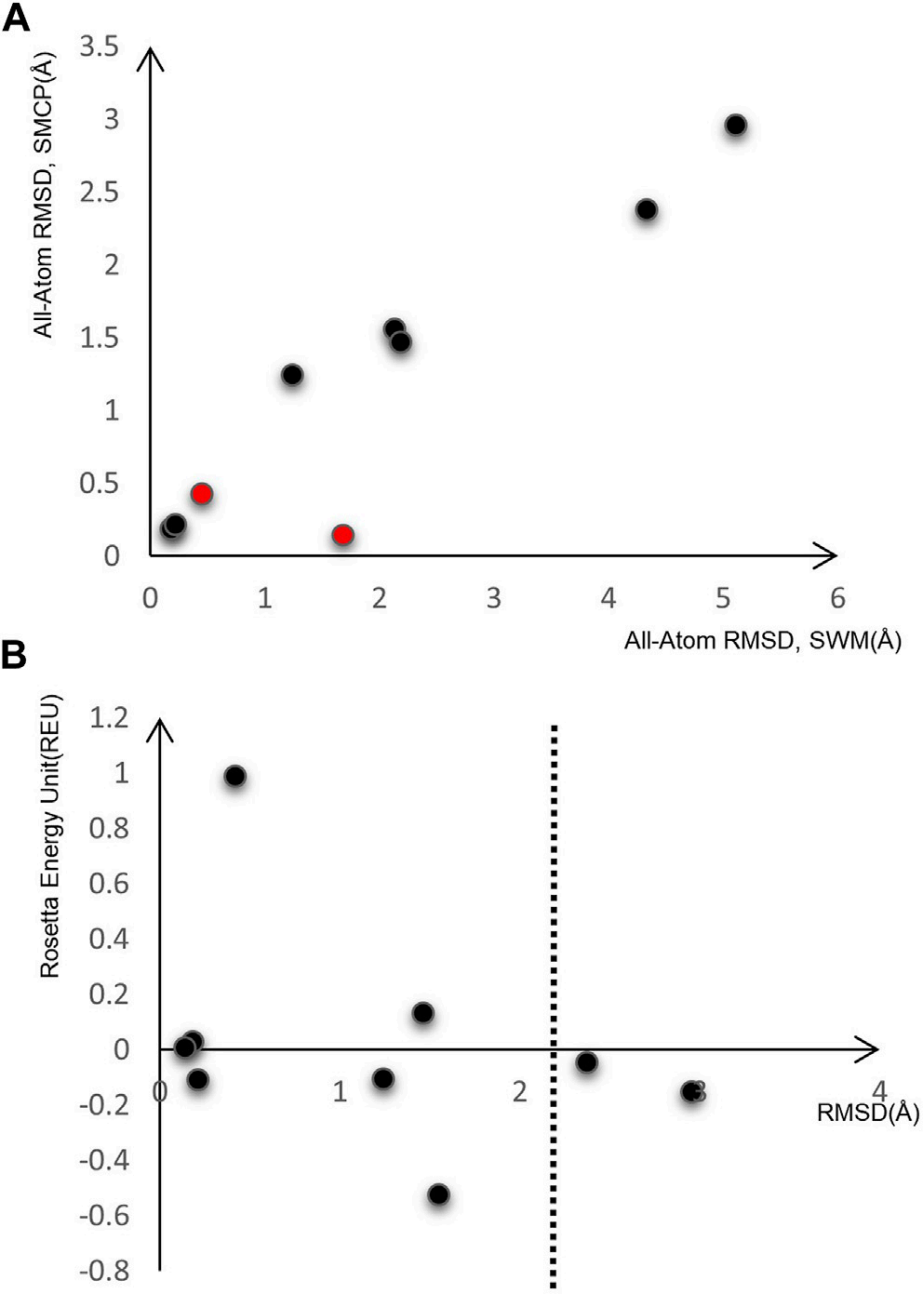
Model on nine RNA motifs. **(A)** Comparison of modeling RMSD between SWM and SMCP. **(B)** SMCP modeling results.

After preliminary testing, we then carried out SMCP modeling and SWM modeling on a group of nine RNA motifs, whose length is between 5–15 nt. Figure 3A shows the result of the SMCP model and SWM model on a benchmark composed of nine RNA motifs, and the abscissa in the figure shows the modeling RMSD value of SWM. Here, the ordinate represents the modeling RMSD value of SMCP, and the RMSD value represents the error between the modeling result and the native structure, which can reflect the modeling accuracy, and its unit is Å. We prefer the RMSD value of SMCP modeling to be minor, which means that the SMCP modeling accuracy is high.

In Figure 3A, the RMSD value of SMCP is lower than that of SWM, indicating that the modeling accuracy of SMCP is higher than that of SWM. We further demonstrate that the parallelization and further judgment of modeling results can improve the modeling accuracy. In the exceptional red cases, the modeling RMSD values obtained by SWM and SMCP are less than 2 Å. In this case, the advantage of SMCP modeling lies in its high modeling integrity. This further shows that the SWCP algorithm can effectively improve the modeling integrity in the modeling process.

As we all know, the lower the structural energy, the stronger the structural stability. Therefore, we expect the conformational energy output after modeling to be low enough. Figure 3B shows the RMSD of nine RNA motifs and their corresponding conformational energy values. Both RMSD and energy values are relatively low, indicating that SMCP is very effective for RNA modeling and can meet the requirements of low energy and high accuracy.

**FIGURE 4:**
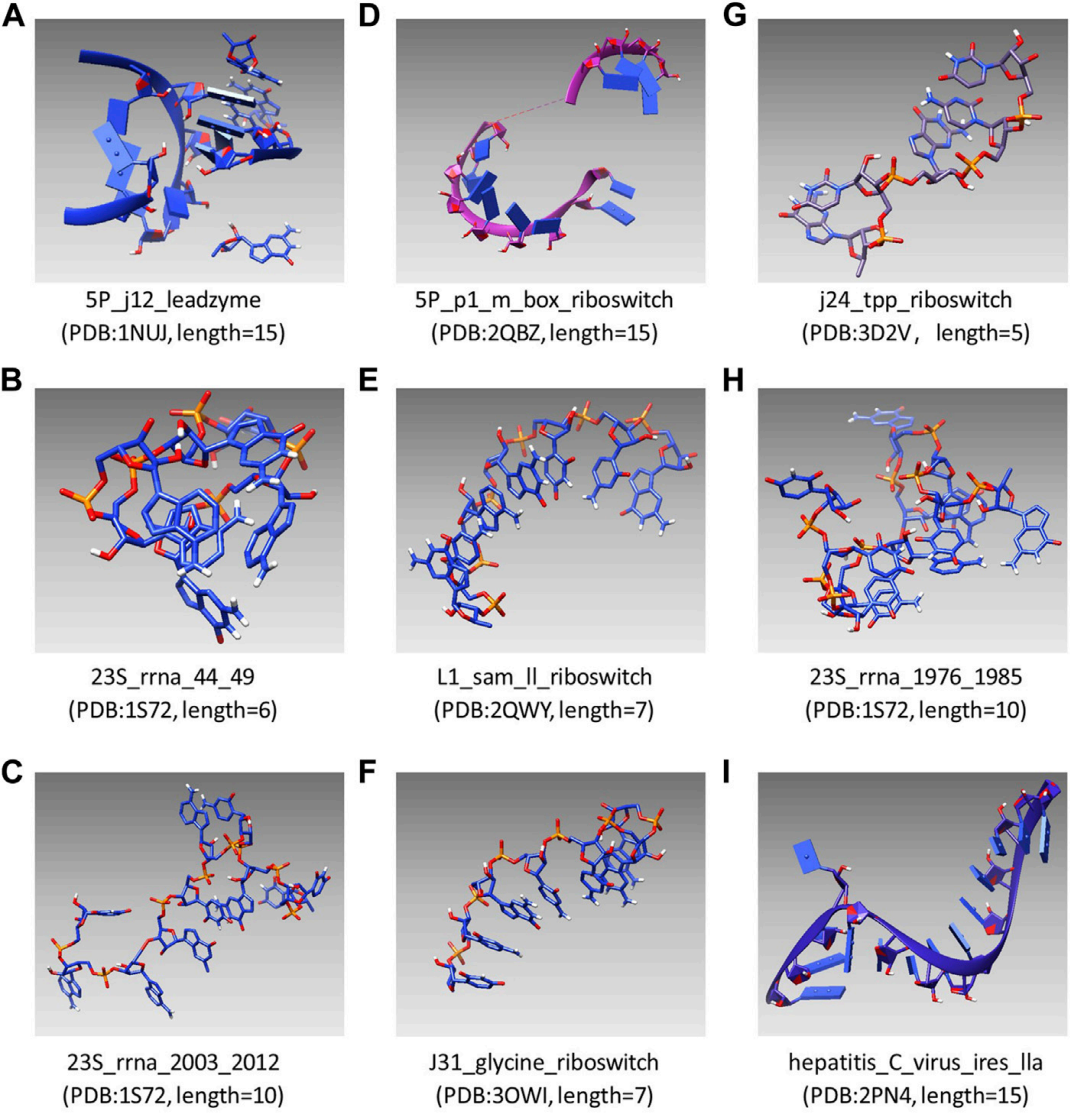
Visual modeling results of the benchmark composed of nine RNA motifs.

UCSF Chimera was first used as an interactive visualization tool for sequence structure analysis (Meng et al., 2006). Later, the tool was used to visualize density maps (Goddard et al., 2007) and nucleic acid (Couch., 2006), which clarified the structure and characteristics of macromolecular components. In recent years, UCSF Chimera has been applied in a sea of fields, which is used to draw the 3D surface of ESCPT protein microscopic data (Le Bars et al., 2019) and used for the drug design of *Mycobacterium tuberculosis* (MTB) (Urooj et al., 2019). In this study, the modeling results of SMCP are visualized by UCSF Chimera, and Figure 4 shows the modeling visualization results of the benchmark composed of nine RNA motifs. In addition, the basic information of nine test motifs, such as name, PDB id, and nucleotide number (length), are also compiled in Figure 4 (A–I).

**FIGURE 5:**
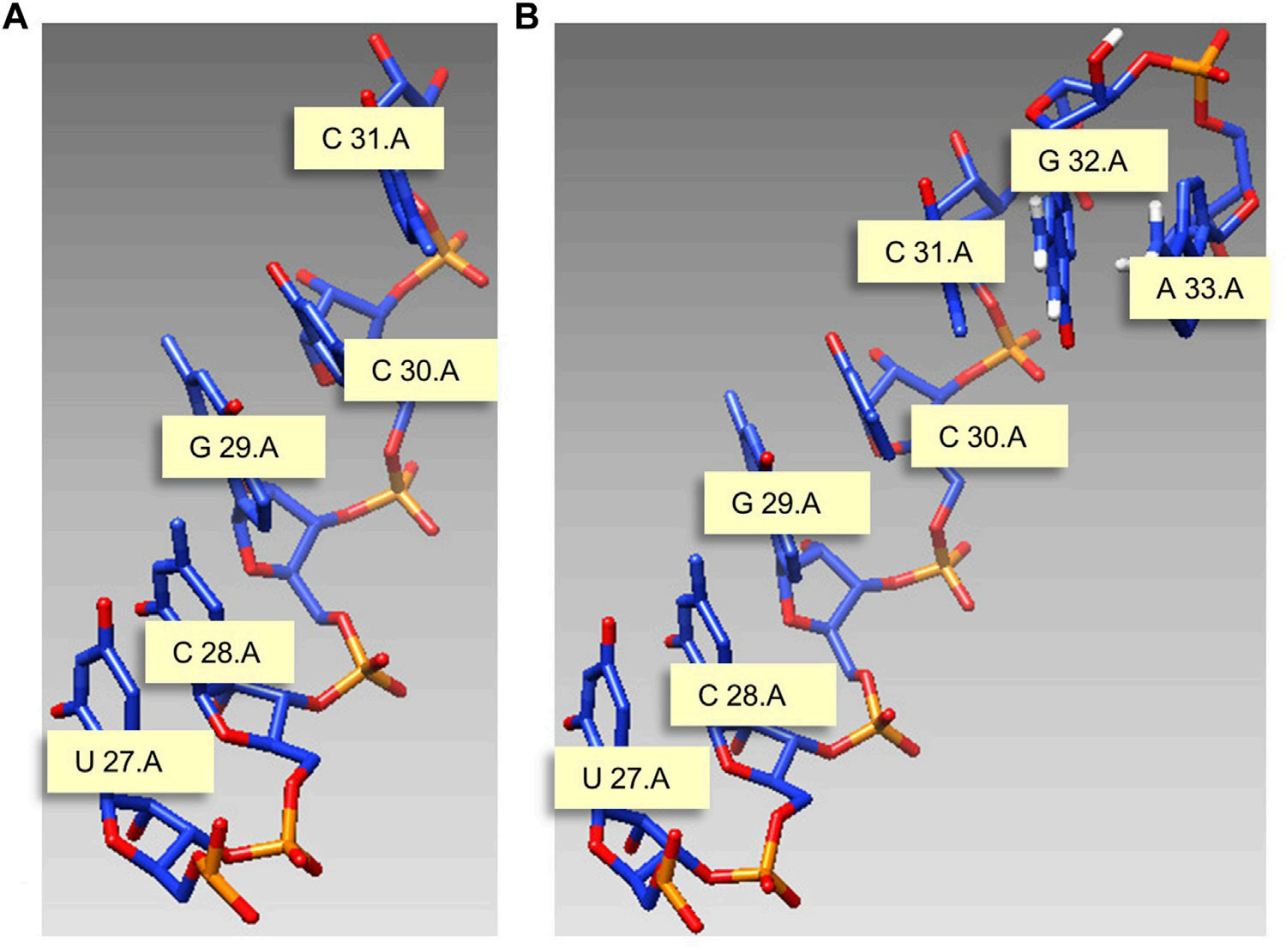
Modeling example with *J31_glycine_riboswitch* as material. **(A)** Start structure. **(B)** Predicted structure. Meaning marked in the figure: U 27.A indicates that the 27th nucleotide in chain A is U.

This study gives a modeling example with J31_glycine_riboswitch as material, and the motif length is 7 nt. Figure 5A is the start structure, and we need to model nucleotides G and A on chain A based on this chain, and the modeling results are shown in Figure 5B.

**FIGURE 6:**
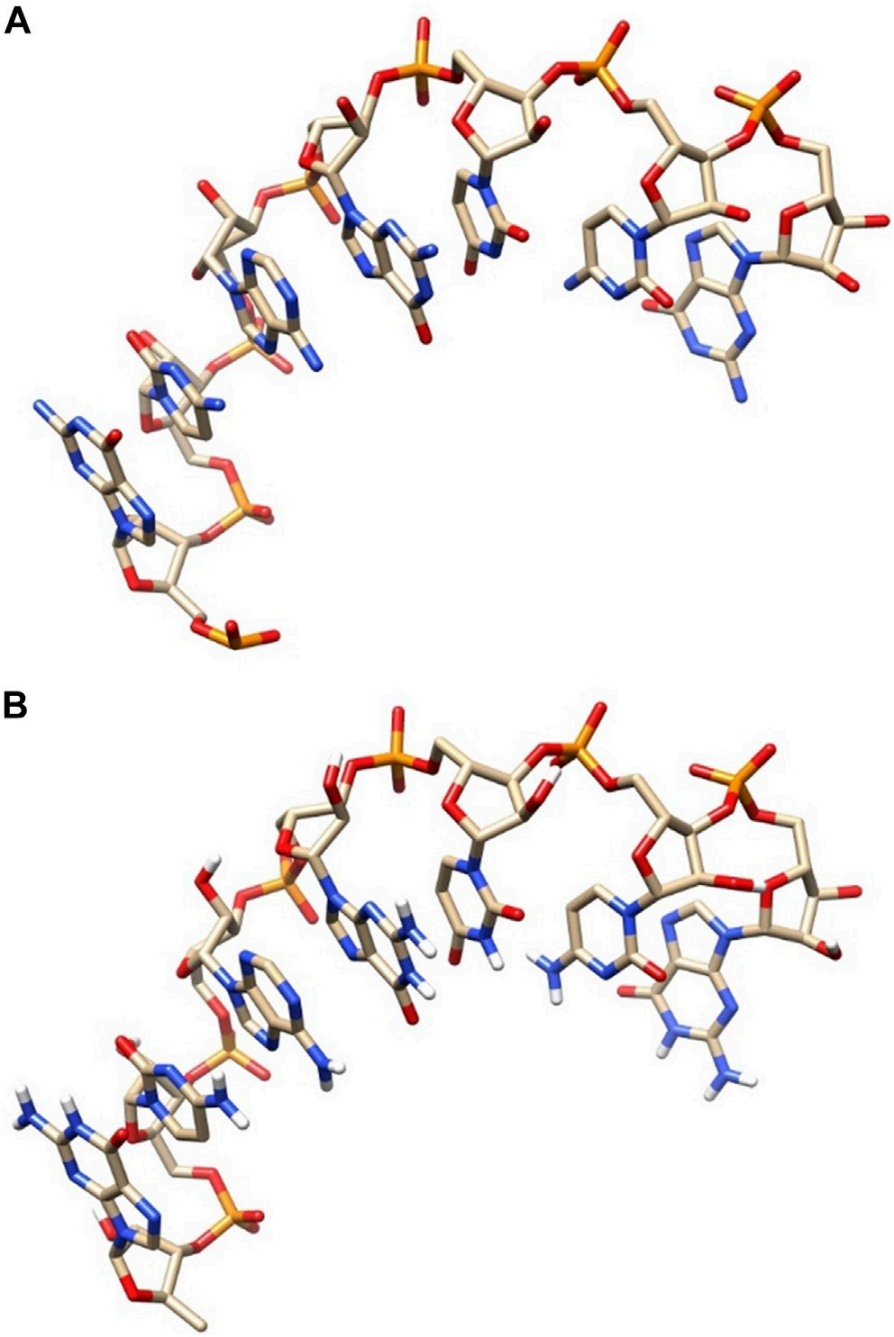
Comparative diagram of the native structure and predicted structure. **(A)** Native structure. **(B)** Predicted structure.

Furthermore, we can compare our model and the native conformation by visual structure comparison. Figure 6 shows the comparison diagram between the modeled conformation and the native conformation of l1_sam_ll_riboswitch (PDB: 2qwy, length: 7). Figure 6A shows the native conformation, and Figure 6B shows the predicted structure. By comparing the two conformations in Figure 6A and Figure 6B, we can see that the difference between the two conformations is very small, and the data also show that the RMSD value is only 0.213Å, which indicates that our structure predicting method has a very high modeling accuracy.

## Conclusion

The sampling method in the existing RNA tertiary structure modeling is still one of the critical factors affecting the modeling accuracy. The Monte Carlo–based sampling method has certain modeling limitations, and the modeling may be incomplete. In view of these limitations, this study proposes a Monte Carlo sampling parallel algorithm and an SMCP algorithm based on the stepwise ansatz assumption. The SMCP algorithm performs two rounds of potential energy evaluation after sampling, detects the modeling results, and finally obtains a better modeling result.

Initialize multiple threads simultaneously, specify the same energy function, use the stepwise ansatz assumption to perform Monte Carlo–based sampling, and then, use the energy function for scoring. After scoring, the algorithm performs a round of potential energy assessments to determine whether the move is reasonable. Then, comprehensively perform two rounds of potential energy evaluation on all threads, select the best modeling results among all threads, and judge the integrity and accuracy of the conformation. Only when it meets the requirements can the conformation be output.

Experiments show that the SMCP algorithm has the following characteristics: 1) High modeling accuracy. Through constant potential energy evaluation, the best conformation is selected. 2) High integrity. Check the modeling results, and the final model includes all nucleotides. 3) High flexibility. The execution times of the algorithm can be set according to actual needs, and the modeling accuracy and modeling time cost can be measured by users.

Although the SMCP algorithm performs well in RNA modeling, it also has certain limitations. The current parallel algorithm uses the same energy function for multiple threads. Still, a single energy function may be insufficient, and RNA modeling is very sensitive to the energy function, so further exploring the algorithm’s optimal energy function is necessary. In addition, multi-threading using different energy functions is also one of our future works.

## Data Availability

The datasets presented in this study can be found in online repositories. The names of the repository/repositories and accession number(s) can be found in the article/Supplementary Material.
